# Histological Assessment of Plasma-Induced Tissue Sublimation Using the Plasma IQ Device: An Ex Vivo Morphometric Study in a Porcine Model

**DOI:** 10.3390/biomedicines14051173

**Published:** 2026-05-21

**Authors:** Paweł Kubik, Wojciech Gruszczyński, Aleksandra Pawłowska, Maciej Malinowski, Brygida Baran, Agnieszka Pawłowska-Kubik, Łukasz Kodłubański, Dariusz Grzanka, Paulina Antosik, Bartłomiej Łukasik

**Affiliations:** 1 K-LAB Badania i Rozwój, 81-312 Gdynia, Poland; wojciech.gruszczynski@k-lab.com.pl (W.G.); aleksandra.pawlowska@k-lab.com.pl (A.P.); maciejmal@gmail.com (M.M.); agnieszkapawl@wp.pl (A.P.-K.); 2 Medical Department, Matex Lab Switzerland SA, 1228 Geneva, Switzerland; brygida.baran@neauvia.com (B.B.); bartlomiej.lukasik@neauvia.com (B.Ł.); 3 Department of Human Rights and Intellectual Property Law, University of Gdansk, 80-309 Gdansk, Poland; lukasz.kod@gmail.com; 4 Department of Clinical Pathomorphology, Faculty of Medicine, Collegium Medicum in Bydgoszcz, Nicolaus Copernicus University in Toruń, 85-094 Bydgoszcz, Poland; d_grzanka@cm.umk.pl; 5 Department of Dermatology and Venerology, Faculty of Medicine, Collegium Medicum in Bydgoszcz, Nicolaus Copernicus University in Toruń, 85-094 Bydgoszcz, Poland; paulina.antosik@cm.umk.pl

**Keywords:** atmospheric plasma, radiofrequency-based plasma generation, tissue sublimation, Plasma IQ, ex vivo histology, morphometric analysis, aesthetic dermatology

## Abstract

**Background**: Minimally invasive aesthetic procedures using atmospheric plasma devices are increasingly applied to improve skin laxity and age-related loss of firmness. These systems generate a localized plasma arc at the tissue surface, enabling controlled and spatially confined tissue interaction; however, quantitative histological data on the extent of plasma-induced tissue effects remain limited. **Materials and Methods**: This ex vivo study evaluated freshly collected porcine kidney, liver, and skeletal muscle tissues (n = 3 per tissue type). Tissue sublimation defects were produced using the Plasma IQ device under conditions representative of standard clinical use, applying two predefined settings (“LOW” and “HIGH”). Immediately after treatment, specimens were fixed in 10% neutral buffered formalin and processed into formalin-fixed paraffin-embedded (FFPE) blocks. Sections were stained with hematoxylin and eosin (H&E), and the diameter and depth of the sublimation zones were measured by light microscopy. **Results**: Plasma IQ exposure consistently produced well-demarcated superficial sublimation defects in all tissues. The HIGH setting increased the diameter of the sublimation zones compared with the LOW setting across all tissue types, whereas the depth differences were smaller and tissue-dependent. Lesions exhibited a characteristic flattened, cone-shaped morphology, with diameter exceeding depth. No histologically detectable collateral damage was observed beyond the immediate sublimation zone. **Conclusions**: Atmospheric plasma treatment induces controlled and spatially confined tissue sublimation with clearly defined histological boundaries and limited penetration depth. These findings provide quantitative histological support for the localized tissue effects of plasma-based devices and their rationale in aesthetic procedures.

## 1. Introduction

Minimally invasive aesthetic procedures employing plasma-based energy systems have gained increasing attention as viable alternatives to surgical intervention for the management of skin laxity, superficial rhytides, and age-related loss of tissue firmness [1,2,3]. These technologies are positioned within the broader landscape of energy-based devices used in aesthetic medicine, alongside radiofrequency (RF), microfocused ultrasound with visualization (MFU-V), and fractional laser platforms, each of which has demonstrated clinical efficacy for non-surgical skin tightening and rejuvenation [4,5,6].

Plasma skin regeneration (PSR) represents a distinct category within this field, utilizing plasma energy rather than light or radiofrequency alone to deliver thermal effects to the skin surface. Unlike laser- or light-based modalities, PSR is not chromophore-dependent and does not vaporize tissue, instead leaving a layer of desiccated but intact epidermis that functions as a natural biologic dressing, thereby supporting wound healing and accelerating recovery [7,8]. PSR has received FDA 510(k) clearance for the treatment of rhytides, superficial skin lesions, actinic keratoses, viral papillomas, and seborrheic keratoses, and has demonstrated additional benefits in the management of dyschromia, photoaging, skin laxity, and acne scarring [7,8,9]. Histological studies in both animal models and human subjects have confirmed that PSR treatment induces significant neo-collagenesis, reduced solar elastosis, and progressive dermal remodeling that may persist beyond one year post-treatment [7,10,11].

The Plasma IQ device (Berger & Kraft Medical Sp. z o.o., Warsaw, Poland), a CE-certified class IIa medical device, represents a further development in plasma-based aesthetic technology. During treatment, when the applicator tip is positioned at an appropriate distance (typically 1–2 mm under standard operating conditions [12]) from the tissue surface, surrounding atmospheric gas becomes ionized, forming a localized plasma arc between the device electrode and the treated tissue. This phenomenon enables focused energy transfer, leading to controlled tissue sublimation, defined as a rapid transition of tissue from a solid to a gaseous state, without direct electrode–skin contact [1,3,13]. Due to the highly localized nature of the plasma arc, the resulting thermal tissue effect is expected to be spatially confined which may contribute to a favorable safety profile when applied in anatomically sensitive regions [1,3,14].

A recent clinical study by Kubik et al. evaluated the clinical efficacy of Plasma IQ for periorbital skin rejuvenation in 30 patients and demonstrated statistically significant improvements in skin elasticity (+22.51% at day 90), alongside a marked increase in eyelid margin–to–fold distance (+49.11% at day 90, *p* < 0.001) [15]. The treatment was well tolerated, with transient erythema (mean 2.77 days) and edema (mean 3.57 days) as the only reported side effects, and both patient and expert satisfaction rates were high. These clinical findings support the therapeutic potential of Plasma IQ; however, quantitative histological characterization of the spatial extent of tissue sublimation and the depth of penetration achieved under different device settings remains limited [14,16].

The controlled superficial microinjury induced by plasma-based treatment is considered a potential trigger for a localized repair response, including activation of dermal fibroblasts and subsequent extracellular matrix (ECM) remodeling processes that may contribute to gradual improvement in skin quality [8,17,18]. In parallel, cold atmospheric plasma (CAP) technologies have attracted increasing interest across medical disciplines due to their diverse biological effects, including generation of reactive oxygen and nitrogen species (RONS) that modulate signaling pathways involved in wound healing, angiogenesis, and cellular proliferation [17,19,20,21]. It is important to note that, unlike CAP technologies whose primary effects are mediated through RONS, the Plasma IQ device produces predominantly thermal in nature, arising from localized energy deposition of the plasma arc; references to CAP literature in this manuscript are provided for contextual comparison of biological mechanisms and should be interpreted in light of this mechanistic distinction. In addition to histological assessment, complementary characterization methods, including immunohistochemistry, cell viability assays, and molecular markers of oxidative stress, have been proposed for more comprehensive evaluation of plasma–tissue interactions [22]. These observations provide a mechanistic rationale for the clinical improvements observed following plasma-based treatments.

Despite the growing clinical popularity of plasma devices, detailed histological characterization of plasma-induced tissue effects remains limited, particularly with regard to objective morphometric assessment of lesion geometry under different device parameters [3,14,16]. Preclinical histological studies comparing plasma/RF systems with conventional monopolar RF devices have shown that plasma-mediated energy delivery produces discrete microscopic zones of thermal effect comparable to standard RF, without evidence of uncontrolled thermal spread [11,23]. However, systematic morphometric data specifically characterizing the diameter and depth of sublimation defects produced by the Plasma IQ device are lacking.

A clear understanding of lesion geometry is essential for optimizing treatment parameters, improving reproducibility, and supporting safe clinical implementation. Therefore, the aim of this study was to evaluate the dimensions of plasma-induced sublimation defects, including diameter and depth, in an ex vivo animal tissue model. Histological assessment was performed on porcine kidney, liver, and skeletal muscle tissues treated under conditions representative of standard clinical use. The experimental procedures were conducted at Centrum Medyczne Doktor Kubik, Gdynia, Poland.

## 2. Materials and Methods

### 2.1. Tissue Material and Study Design

The study was conducted on ex vivo fragments of freshly collected porcine kidney, liver, and skeletal muscle tissues, selected to represent tissues with different hydration and structural properties. These tissues were selected as commercially available, standardized specimens suitable for controlled ex vivo experimentation and used in prior energy-device research [11,23]. Porcine skin, the primary clinical target tissue, was not feasible for this pilot study; skin-specific ex vivo studies are planned to validate the present findings. The selection of tissue models was furthermore governed by the requirements of the FDA 510(k) regulatory submission for the Plasma IQ device, within which porcine kidney, liver, and skeletal muscle were specified as test substrates to characterize energy delivery geometry across tissues of varying impedance and hydration, properties relevant to electrical safety assessment. Complementary histological data from human periorbital skin are available in the companion clinical study [15]. The specimens were purchased commercially, and no animal procedures were performed by the investigators; therefore, ethical approval was not required. Three independent samples were analyzed for each tissue type. Following delivery, tissues were processed immediately under controlled conditions to minimize dehydration and preserve morphology.

### 2.2. Plasma Device and Experimental Conditions

Experiments were performed using the Plasma IQ device (Berger & Kraft Medical Sp. z o.o., Warsaw, Poland), a CE-certified class IIa medical device for plasma-assisted tissue sublimation, equipped with a standard manufacturer-provided treatment electrode. According to the manufacturer’s technical documentation, the generator operates at a frequency of 40 kHz (low frequency [LF] band per ITU classification; 30–300 kHz), with a maximum power output of up to 5 W in continuous mode [12]. All procedures were carried out in an incubator maintained at 36 °C (±0.5 °C) to ensure consistent thermal conditions during exposure.

### 2.3. Plasma Treatment Protocol

Tissue fragments were positioned on a flat surface. All procedures were performed in non-contact mode; the active electrode tip was maintained at a distance from the tissue surface without direct contact, consistent with the plasma arc formation principle [12]. In the aesthetic mode used in this study, the device operates without a neutral (return) electrode, functioning as a high-voltage, low-power non-contact discharge system in which the plasma arc is formed between the active electrode tip and the tissue surface acting as the grounded reference. Plasma treatment was applied in aesthetic mode at two manufacturer-defined power settings. In the LOW setting, the source output voltage is 650 VAC (RMS) with the electrode voltage reaching 950 VAC (RMS); in the HIGH setting, the source voltage is 950 VAC (RMS) with the electrode voltage reaching approximately 1100 VAC (RMS) [12]. Maximum device power output is 5 W at rated impedance of 54 kΩ [24]. Each tissue site received a single plasma application; the duration of each application was approximately 1 s per setting. Immediately after treatment, all samples were transferred for fixation.

### 2.4. Fixation and Processing for Histological Evaluation

Specimens were fixed in 10% neutral buffered formalin (pH 7.2) and subsequently processed at the Department of Clinical Pathology, Collegium Medicum in Bydgoszcz, Nicolaus Copernicus University in Toruń, Poland. Formalin-fixed tissues were processed using routine protocols, including dehydration, clearing, and paraffin embedding, on a Thermo Scientific™ Excelsior™ AS automated tissue processor (Thermo Fisher Scientific, Runcorn, UK), to obtain formalin-fixed paraffin-embedded (FFPE) tissue blocks. 

### 2.5. Microtome Sectioning and Slide Preparation

FFPE blocks were sectioned at 5 μm using a manual rotary microtome (Accu-Cut, Sakura Finetek, Torrance, CA, USA). Sections were mounted on adhesive glass slides (Superfrost Plus; Menzel-Gläser, Braunschweig, Germany) to improve tissue adherence during staining.

### 2.6. Hematoxylin and Eosin Staining

Routine hematoxylin and eosin (H&E) staining was performed to evaluate general morphology and tissue architecture. Slides were deparaffinized, rehydrated, stained with hematoxylin and eosin, dehydrated, cleared, and cover-slipped according to standard procedures.

### 2.7. Histological Assessment and Morphometric Measurements

Histological examination was performed using an ECLIPSE E800 light microscope (Nikon Instruments Europe, Amsterdam, The Netherlands) at ×10 magnification. All slides were assessed independently by two pathologists. The diameter and depth of the plasma-induced sublimation areas were measured, and representative microphotographs were obtained. All H&E-stained sections used in the morphometric analysis are provided in the Supplementary Materials (Figures S1–S3).

### 2.8. Statistical Analysis

Given the pilot nature of this study (n = 3 per tissue and setting), differences in sublimation diameter and depth between LOW and HIGH settings were assessed using the Mann–Whitney U test with exact *p*-values. The non-parametric approach was selected because normality cannot be verified at this sample size. Effect sizes were quantified as the rank-biserial correlation coefficient (r), where |r| = 1.000 indicates complete separation between groups. With n = 3 per group, the minimum achievable two-sided *p*-value is 0.100; all comparisons are therefore exploratory.

## 3. Results

**Figure 1:**
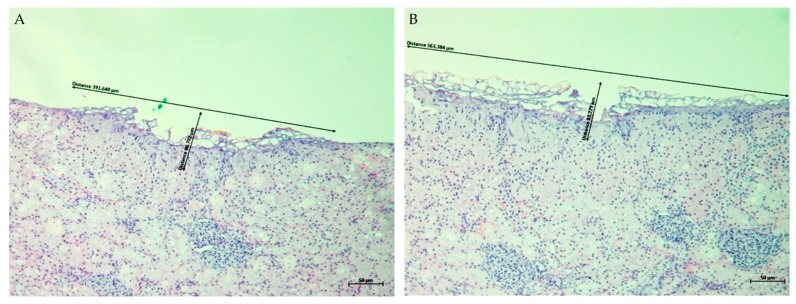
Representative H&E-stained sections of porcine kidney tissue after Plasma IQ treatment (×10). (**A**) LOW setting: a well-demarcated superficial sublimation defect with a lateral diameter of 391.640 µm and a depth of 90.772 µm. (**B**) HIGH setting: sublimation defect with a lateral diameter of 563.384 µm and a depth of 89.279 µm. Black arrows with measurement annotations indicate the diameter and depth of the sublimation area. Scale bar = 50 µm. Note: commas in on-image labels denote decimal separators.

**Figure 2:**
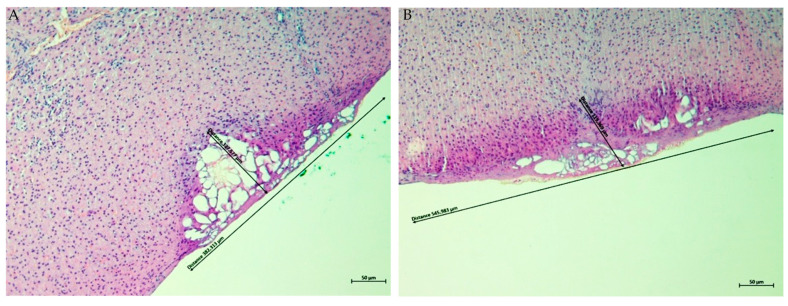
Representative H&E-stained sections of porcine liver tissue after Plasma IQ treatment (×10). (**A**) LOW setting: a well-demarcated superficial sublimation defect with a lateral diameter of 382.313 µm and a depth of 127.577 µm. (**B**) HIGH setting: sublimation defect with a lateral diameter of 545.983 µm and a depth of 119.369 µm. Black arrows with measurement annotations indicate the diameter and depth of the sublimation area. Scale bar = 50 µm. Note: commas in on-image labels denote decimal separators.

**Figure 3:**
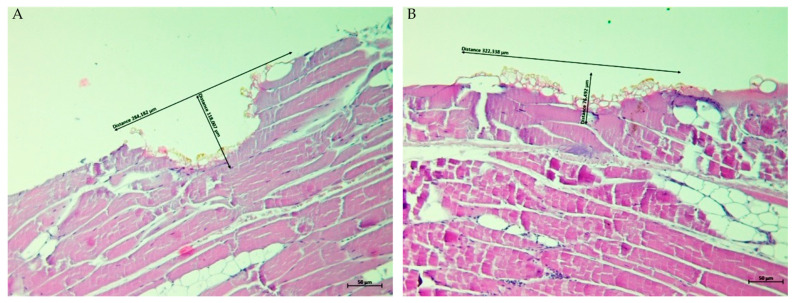
Representative H&E-stained sections of porcine skeletal muscle tissue after Plasma IQ treatment (×10). (**A**) LOW setting: a well-demarcated superficial sublimation defect with a lateral diameter of 284.182 µm and a depth of 118.007 µm. (**B**) HIGH setting: sublimation defect with a lateral diameter of 322.338 µm and a depth of 76.492 µm. Black arrows with measurement annotations indicate the diameter and depth of the sublimation area. Scale bar = 50 µm. Note: commas in on-image labels denote decimal separators.

Histological examination of ex vivo porcine kidney, liver, and skeletal muscle tissues treated with the Plasma IQ device confirmed the presence of localized surface defects consistent with plasma-induced tissue sublimation. In all specimens, treatment resulted in a clearly demarcated superficial crater with measurable diameter and depth. Representative H&E-stained sections obtained at ×10 magnification are shown in Figure 1, Figure 2 and Figure 3, and quantitative measurements are summarized in Table 1.

In kidney tissue, both LOW and HIGH settings produced distinct sublimation areas (Figure 1). The mean sublimation diameter was higher in the HIGH setting compared with the LOW setting (567.557 ± 10.040 μm, r = −0.333, vs. 445.130 ± 133.782 μm). In contrast, the LOW setting resulted in greater average sublimation depth than the HIGH setting (115.831 ± 56.308 μm, r = +0.111, vs. 92.313 ± 13.482 μm). Notably, variability in measured diameter and depth was higher under the LOW setting.

In liver tissue, plasma exposure generated a superficial defect with adjacent thermally affected margins (Figure 2). The HIGH setting produced a substantially larger mean diameter of sublimation compared to the LOW setting (537.875 ± 20.800 μm, r = −1.000, vs. 332.124 ± 35.523 μm). The depth of tissue effect was comparable between settings, measuring 109.507 ± 7.634 μm for HIGH (r = +0.111) and 112.140 ± 17.387 μm for LOW.

In skeletal muscle, plasma-induced sublimation defects were observed under both treatment settings (Figure 3). The mean sublimation diameter increased from 256.954 ± 42.575 μm in the LOW setting to 368.967 ± 72.295 μm in the HIGH setting (r = −1.000). The corresponding mean depths were 86.582 ± 25.534 μm (LOW) and 75.650 ± 7.368 μm (HIGH; r = +0.111), respectively.

Overall, the HIGH setting consistently increased the diameter of the sublimation defect across all tested tissues, whereas changes in lesion depth were less pronounced and varied depending on tissue type. Complete stochastic dominance of HIGH over LOW (r = −1.000) was observed for diameter in liver and muscle; kidney showed the same directional trend with weaker effect (r = −0.333). No consistent directional effect was found for depth (|r| ≤ 0.333). All comparisons are exploratory given the pilot sample size.

## 4. Discussion

Atmospheric plasma devices used in aesthetic medicine generate plasma under ambient pressure through ionization of air between the electrode tip and the tissue surface, enabling highly localized energy delivery and controlled superficial tissue interaction [1,3,13]. This mechanism has been increasingly adopted in clinical practice due to the possibility of inducing precise microinjury with limited lateral thermal spread, a factor considered critical for procedural safety and predictability [1,3,14]. Although various plasma systems differ in technical implementation, including differences in ionization gas, electrode configuration, and energy delivery mode, the central concept of confining tissue effects to a small treatment area remains a key rationale for their aesthetic application [3,7,17].

In the present ex vivo study, Plasma IQ treatment consistently produced well-defined superficial sublimation defects in porcine kidney, liver, and skeletal muscle tissues, as confirmed by histological evaluation (Figure 1, Figure 2 and Figure 3). The plasma-induced lesions demonstrated crater-like defects with a flattened cone-shaped morphology, in which the lateral dimension exceeded the depth of penetration. This geometric pattern supports the concept of a predominantly superficial tissue effect. Similar histological characteristics and reproducible microthermal injury patterns have been described for Plasma IQ in skin tissue models, where focal microthermal wounds were shown to be dependent primarily on device power settings [14]. These observations suggest that, despite tissue-specific differences, plasma-induced lesions tend to preserve a confined injury profile that can be modulated through treatment parameters.

Quantitative morphometric assessment demonstrated that device settings significantly influenced the extent of tissue sublimation (Table 1). Across all examined tissues, the HIGH setting produced larger mean sublimation diameters compared with the LOW setting, indicating that higher output expands the lateral extent of tissue involvement. In contrast, changes in depth were less pronounced and appeared tissue-dependent, with the greatest measured depth being 193.820 μm in kidney tissue at the LOW setting. The calculated diameter-to-depth ratios were higher under HIGH settings across all tissues, suggesting that increased energy delivery results primarily in wider lesions with proportionally limited depth. Such a pattern may be clinically advantageous, as it may allow broader superficial effect while reducing the likelihood of deep unintended injury, supporting the rationale for plasma devices as precision tools in aesthetic procedures performed in anatomically sensitive regions [1,3,14,15].

These morphometric findings are consistent with preclinical data from other plasma and RF systems. Ruff reported that both plasma/RF and monopolar RF platforms produced discrete zones of thermal effect in the porcine dermis and fibroseptal network, with no significant differences in average or maximum depths of thermal injury between systems [23]. Similarly, Holcomb and Schucker compared helium and nitrogen plasma skin regeneration in a porcine model and demonstrated that thermal injury depth is primarily determined by energy density and number of passes, with both platforms producing well-demarcated lesions confined to the superficial dermis [11]. In a related context, Wootten et al. evaluated radiofrequency microneedling in porcine skin and showed that thermal coagulation zones exhibit a layered, flame-like architecture with the extent of injury dependent on specific device parameters [24]. These collective observations reinforce the principle that energy-based devices, when appropriately calibrated, produce predictable and spatially confined tissue effects, a finding confirmed in the present study for the Plasma IQ system.

Notable tissue-dependent differences were observed in lesion dimensions, with liver and kidney generally demonstrating larger lesion diameters compared with skeletal muscle. This variability may be attributed to differences in hydration, microstructure, and baseline tissue composition, all of which can influence plasma–tissue interaction [3,17]. Furthermore, the LOW setting produced greater inter-sample variability in kidney tissue compared with the HIGH setting, whereas HIGH settings were generally more reproducible. This may indicate that at lower energy output, the tissue response is more sensitive to local microenvironmental factors, while higher output settings generate a more consistent effect. Similar observations regarding the relationship between energy output and lesion reproducibility have been made in the context of fractional laser technologies [25]. These tissue-specific findings also have implications for transferability to skin as the primary clinical target. The dermis and epidermis differ substantially from the investigated tissues in collagen density, layered architecture, and hydration; these properties are expected to influence thermal diffusion and may result in different lesion geometries for equivalent energy delivery. The qualitative pattern of confined sublimation with diameter exceeding depth, consistent across all three structurally distinct tissues, is expected to be broadly preserved in skin as supported by existing porcine skin data [11,14] and with clinical histological data from human periorbital skin reported by Kubik et al. [15].

Although the present experiments were performed on non-cutaneous tissues, the confined lesion morphology and limited penetration depth provide relevant structural context for clinical plasma applications aimed at controlled superficial injury [26,27]. In the clinical study by Kubik et al., Plasma IQ treatment of the periorbital region produced micro-lesions with a mean diameter of approximately 418 μm and a mean depth of approximately 99 μm, with carbon crusts typically detaching within approximately 7 days [15]. These clinical lesion dimensions are broadly consistent with the morphometric ranges observed in the present ex vivo study, particularly for the LOW setting in kidney and liver tissues. The concordance between ex vivo and clinical measurements supports the translational relevance of the present experimental model and confirms that Plasma IQ produces controlled, superficial microinjuries both in experimental and clinical settings.

The present study was not designed to assess biological repair mechanisms; however, the observed microinjury pattern supports the hypothesis that plasma-induced sublimation may act as a controlled stimulus initiating tissue repair and remodeling. In aesthetic medicine, improvement of skin texture and tightening effects is commonly associated with collagen remodeling processes triggered by controlled thermal injury. Histological studies following PSR treatment have demonstrated significant neo-collagenesis and reduction in elastosis in the upper dermis, with a mean depth of new collagen formation of 72.3 μm following low-energy protocols [7,10,11]. Other energy-based modalities, such as monopolar radiofrequency and microfocused ultrasound, have also demonstrated efficacy in dermal tightening through similar wound-healing mechanisms [4,5]. Additionally, cold atmospheric plasma has been proposed as a promising modality in dermo-cosmetic applications, with reported effects on skin biology mediated by reactive species signaling and controlled surface interactions [17,19,20]. These biological considerations provide context for interpreting the controlled injury patterns observed in our morphometric analysis. It should be acknowledged that plasma treatment also generates RONS whose tissue penetration depth may not correspond directly to the thermally damaged zone assessed histologically; RONS may contribute to broader biological responses, including fibroblast activation and angiogenesis that were beyond the scope of the present morphometric investigation [17,18,19].

The results of this study also carry implications for treatment optimization. The finding that increased energy primarily expands lesion width rather than depth suggests that clinicians may modulate the HIGH setting to achieve broader surface coverage without proportionally increasing the risk of deep tissue injury. Conversely, the LOW setting may be preferred in regions where minimal lateral spread is desired, although the somewhat greater variability observed at lower energy should be taken into account. Combined with the clinical tolerability data from Kubik et al., where 76.7% of patients rated the procedure as “barely felt” or “moderately painful” [15], these findings support the clinical applicability of Plasma IQ in anatomically sensitive areas such as the periorbital region.

### Limitations

This ex vivo study does not capture the biological complexity of living tissue, and the investigated porcine kidney, liver, and skeletal muscle tissues do not directly represent human skin, the primary target of aesthetic plasma procedures. The limited sample size (n = 3 per tissue and setting) and use of routine H&E staining restrict statistical inference and may underestimate subtle tissue alterations. Due to the pilot sample size (n = 3 per group), the minimum achievable two-sided *p*-value for the Mann–Whitney U test is 0.100, precluding conventional significance thresholds; all statistical analyses are exploratory. Plasma exposure was performed using manufacturer-defined clinical settings, which may limit reproducibility across different experimental conditions. Additionally, tissue-level power density and electromagnetic field distribution were not independently measured, and real-time temperature monitoring was not conducted. Leakage current values during tissue treatment were not recorded; future studies incorporating real-time electrical monitoring would provide a more complete characterization of device–tissue interactions. Treatment duration per application was not formally standardized, representing a further methodological limitation. Future studies should employ porcine or human skin ex vivo models, larger sample sizes, and standardized exposure parameters to enable definitive conclusions and direct clinical extrapolation.

## 5. Conclusions

Plasma IQ microcurrent radiofrequency treatment induces reproducible and well-confined tissue sublimation with clearly defined histological boundaries and limited penetration depth. The HIGH setting consistently produces wider sublimation defects across all tissue types, while depth remains relatively stable and tissue-dependent. No histologically detectable collateral damage was observed beyond the immediate sublimation zone. These findings provide quantitative histological evidence supporting the controlled nature of plasma-induced tissue effects and offer a structural rationale for the clinical efficacy and safety observed in periorbital rejuvenation studies. Future research should incorporate skin-specific ex vivo and in vivo models, larger sample sizes, standardized reporting of exposure parameters, direct comparisons with established energy-based modalities, and long-term evaluation of remodeling outcomes and safety profiles.

## Figures and Tables

**Table 1:** Morphometric measurements of plasma-induced tissue sublimation in porcine kidney, liver, and skeletal muscle tissues treated with Plasma IQ at LOW and HIGH settings.

Tissue	Setting	Parameter (µm)	Sample 1	Sample 2	Sample 3	Mean	SD
Kidney	LOW	Diameter	629.040	391.640	314.711	445.130	133.782
Kidney	LOW	Depth	193.820	90.772	62.901	115.831	56.308
Kidney	HIGH	Diameter	563.384	557.891	581.397	567.557	10.040
Kidney	HIGH	Depth	89.279	110.131	77.528	92.313	13.482
Liver	LOW	Diameter	382.313	308.929	305.131	332.124	35.523
Liver	LOW	Depth	127.577	87.847	120.996	112.140	17.387
Liver	HIGH	Diameter	545.983	509.333	558.310	537.875	20.800
Liver	HIGH	Depth	119.369	108.379	100.772	109.507	7.634
Muscle	LOW	Diameter	289.848	284.182	196.833	256.954	42.575
Muscle	LOW	Depth	86.277	118.007	55.463	86.582	25.534
Muscle	HIGH	Diameter	322.338	471.080	313.482	368.967	72.295
Muscle	HIGH	Depth	76.492	84.223	66.234	75.650	7.368

Values represent the diameter and depth of the sublimation defect measured on H&E-stained sections (n = 3 per tissue and setting). Data are presented as individual measurements and summarized as mean and standard deviation (SD). All values are given in micrometers (µm).

## Data Availability

The data presented in this study are available on request from the corresponding author. The data are not publicly available due to privacy restrictions.

## Supplementary Materials

The following supporting information can be downloaded at: https://www.mdpi.com/article/10.3390/biomedicines14051173/s1, Figure S1: Representative H&E-stained sections of porcine kidney tissue after Plasma IQ treatment (×10). (A) LOW setting: representative sublimation defects with lateral diameters of 629.040 µm (top panel) and 314.711 µm (bottom panel), and depths of 193.820 µm and 62.901 µm, respectively. (B) HIGH setting: representative sublimation defects with lateral diameters of 581.397 µm (top panel) and 557.891 µm (bottom panel), and depths of 77.528 µm and 110.131 µm, respectively. Black arrows with measurement annotations indicate the diameter and depth of the sublimation area. Scale bar = 50 µm (100 µm in the top panel of A). Note: commas in on-image labels denote decimal separators. Figure S2: Representative H&E-stained sections of porcine liver tissue after Plasma IQ treatment (×10). (A) LOW setting: representative sublimation defects with lateral diameters of 308.929 µm (top panel) and 305.131 µm (bottom panel), and depths of 87.847 µm and 120.996 µm, respectively. (B) HIGH setting: representative sublimation defects with lateral diameters of 509.333 µm (top panel) and 558.310 µm (bottom panel), and depths of 108.379 µm and 100.772 µm, respectively. Black arrows with measurement annotations indicate the diameter and depth of the sublimation area. Scale bar = 50 µm. Note: commas in on-image labels denote decimal separators. Figure S3: Representative H&E-stained sections of porcine skeletal muscle tissue after Plasma IQ treatment (×10). (A) LOW setting: representative sublimation defects with lateral diameters of 289.848 µm (top panel) and 196.833 µm (bottom panel), and depths of 86.277 µm and 55.463 µm, respectively. (B) HIGH setting: representative sublimation defects with lateral diameters of 471.080 µm (top panel) and 313.482 µm (bottom panel), and depths of 84.223 µm and 66.234 µm, respectively. Black arrows with measurement annotations indicate the diameter and depth of the sublimation area. Scale bar = 50 µm. Note: commas in on-image labels denote decimal separators.
