# Supplementary material for: Histological Assessment of Plasma-Induced Tissue Sublimation Using the Plasma IQ Device: An Ex Vivo Morphometric Study in a Porcine Model
Source: Biomedicines. 2026 May 21;14(5):1173. doi: 10.3390/biomedicines14051173 (PMC13204154; doi:10.3390/biomedicines14051173)
Supplement: Supplementary file 1 [file biomedicines-14-01173-s001.zip › biomedicines-4291209-supplementary.pdf]

Supplementary Material

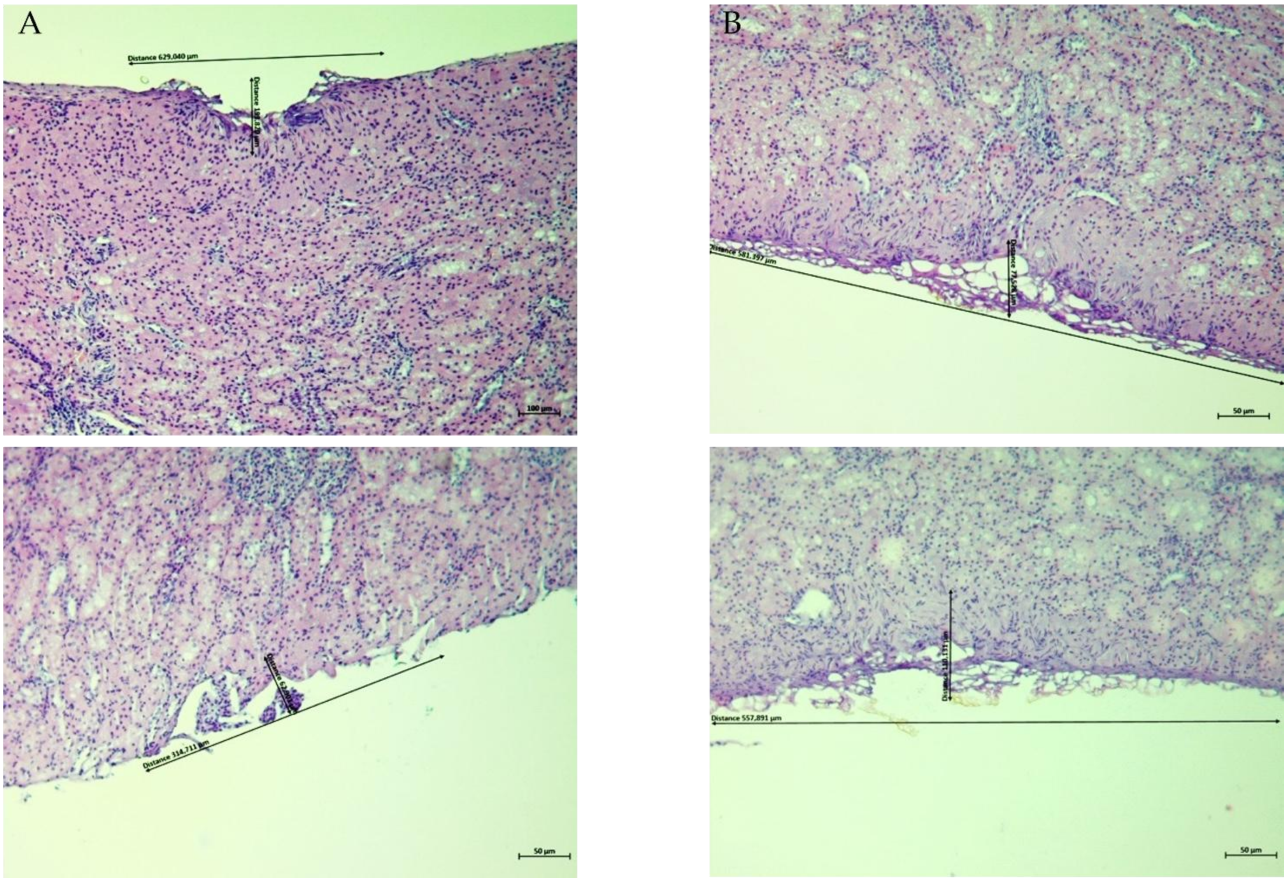

Figure S1. Representative H&E-stained sections of porcine kidney tissue after Plasma IQ treatment (×10). **(A)** LOW setting: representative sublimation defects with lateral diameters of 629.040 µm (top panel) and 314.711 µm (bottom panel), and depths of 193.820 µm and 62.901 µm, respectively. **(B)** HIGH setting: representative sublimation defects with lateral diameters of 581.397 µm (top panel) and 557.891 µm (bottom panel), and depths of 77.528 µm and 110.131 µm, respectively. Black arrows with measurement annotations indicate the diameter and depth of the sublimation area. Scale bar = 50 µm (100 µm in the top panel of A). *Note: commas in on-image labels denote decimal separators*

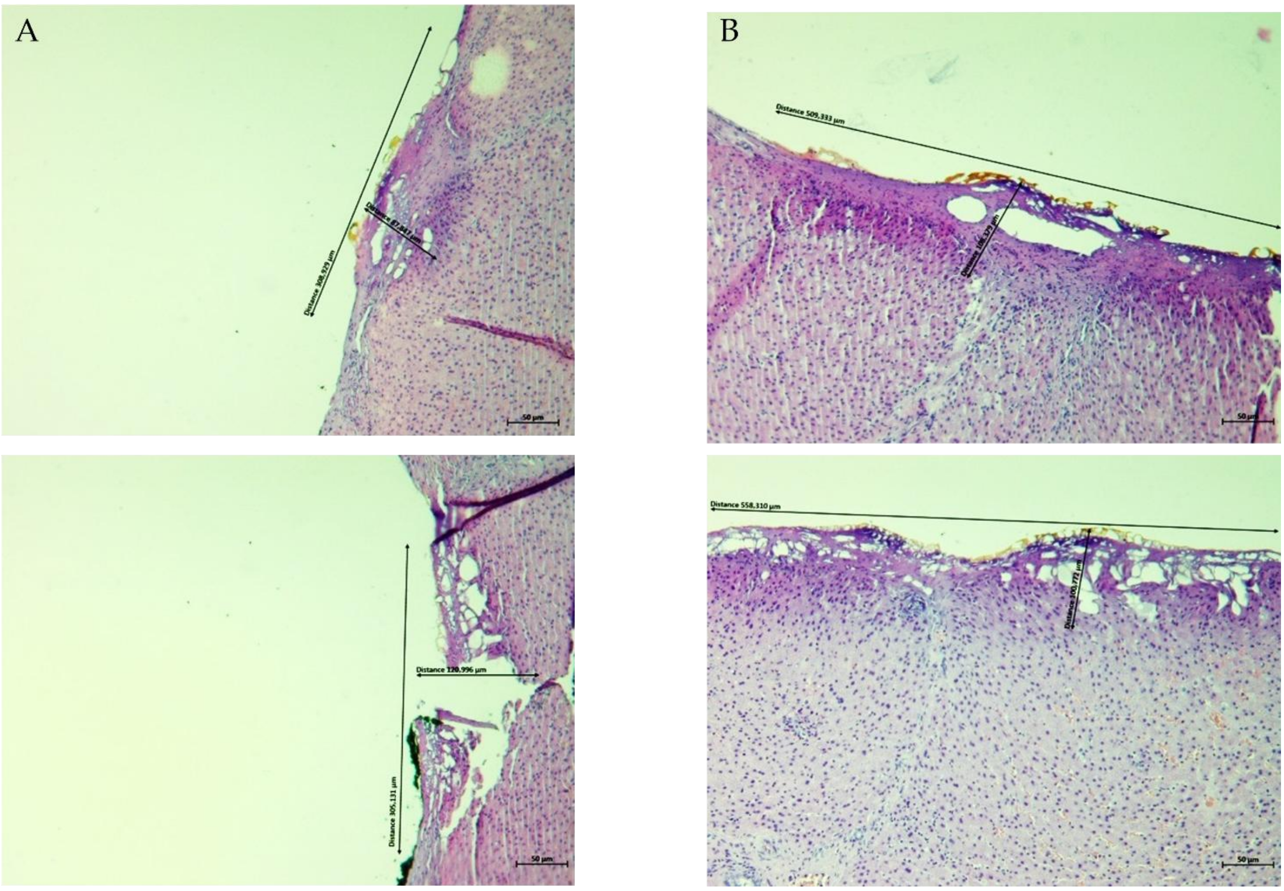

Figure S2. Representative H&E-stained sections of porcine liver tissue after Plasma IQ treatment (×10). **(A)** LOW setting: representative sublimation defects with lateral diameters of 308.929 µm (top panel) and 305.131 µm (bottom panel), and depths of 87.847 µm and 120.996 µm, respectively. **(B)** HIGH setting: representative sublimation defects with lateral diameters of 509.333 µm (top panel) and 558.310 µm (bottom panel), and depths of 108.379 µm and 100.772 µm, respectively. Black arrows with measurement annotations indicate the diameter and depth of the sublimation area. Scale bar = 50 µm. *Note: commas in on-image labels denote decimal separators.*

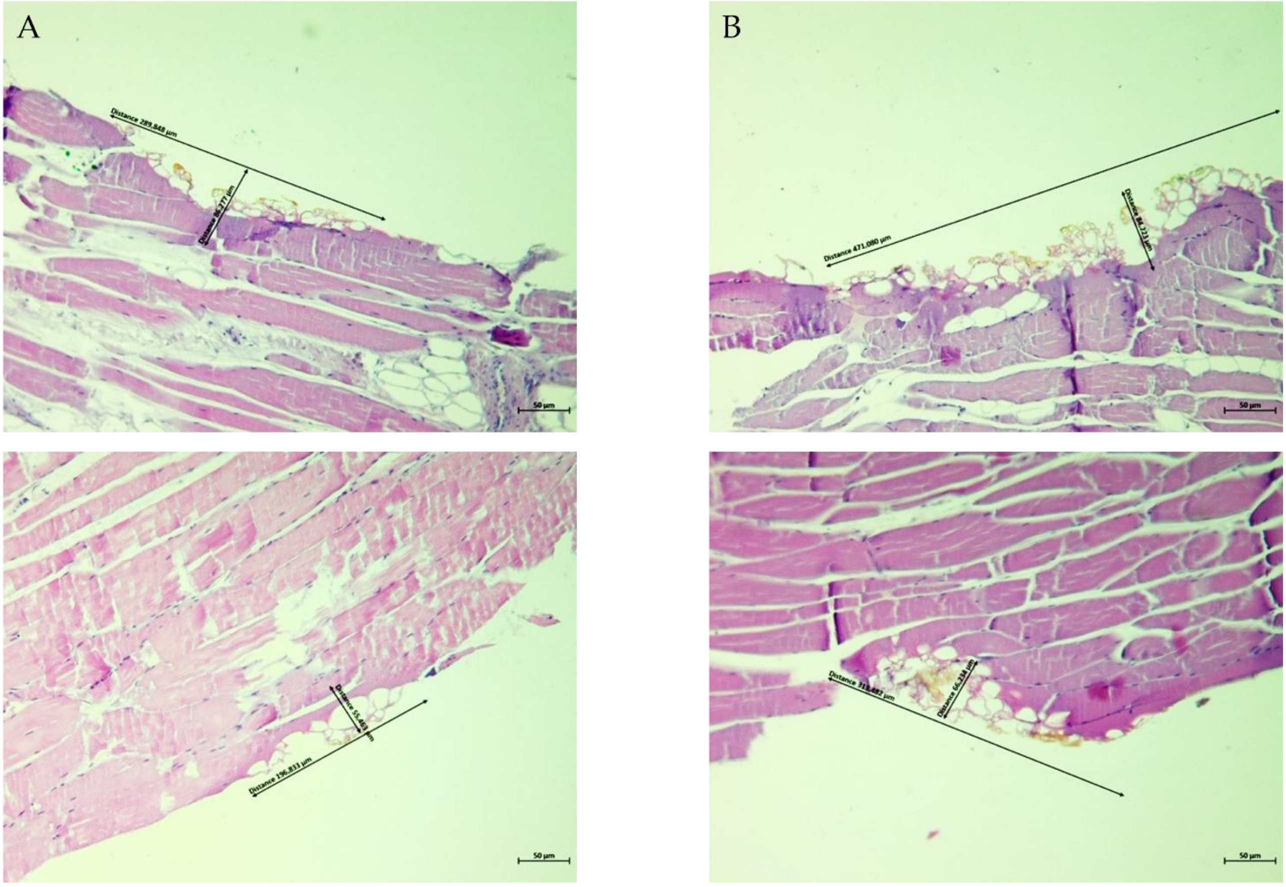

Figure S3. Representative H&E-stained sections of porcine skeletal muscle tissue after Plasma IQ treatment (×10). **(A)** LOW setting: representative sublimation defects with lateral diameters of 289.848 µm (top panel) and 196.833 µm (bottom panel), and depths of 86.277 µm and 55.463 µm, respectively. **(B)** HIGH setting: representative sublimation defects with lateral diameters of 471.080 µm (top panel) and 313.482 µm (bottom panel), and depths of 84.223 µm and 66.234 µm, respectively. Black arrows with measurement annotations indicate the diameter and depth of the sublimation area. Scale bar = 50 µm. *Note: commas in on-image labels denote decimal separators.*
